# Overcoming bioprocess bottlenecks in the large-scale expansion of high-quality hiPSC aggregates in vertical-wheel stirred suspension bioreactors

**DOI:** 10.1186/s13287-020-02109-4

**Published:** 2021-01-13

**Authors:** Breanna S. Borys, Tiffany Dang, Tania So, Leili Rohani, Tamas Revay, Tylor Walsh, Madalynn Thompson, Bob Argiropoulos, Derrick E. Rancourt, Sunghoon Jung, Yas Hashimura, Brian Lee, Michael S. Kallos

**Affiliations:** 1 Pharmaceutical Production Research Facility, Schulich School of Engineering, University of Calgary, 2500 University Dr. NW, Calgary, AB T2N 1N4 Canada; 2 Biomedical Engineering Graduate Program, University of Calgary, 2500 University Dr. NW, Calgary, AB T2N 1N4 Canada; 3 Department of Chemical and Petroleum Engineering, Schulich School of Engineering, University of Calgary, 2500 University Dr. NW, Calgary, AB T2N 1N4 Canada; 4 Department of Biochemistry and Molecular Biology, Cumming School of Medicine, University of Calgary, 3330 Hospital Dr. NW, Calgary, AB T2N 4N1 Canada; 5 Department of Medical Genetics, Alberta Health Services, Alberta Children’s Hospital, 28 Oki Drive, Calgary, AB T3B 6A8 Canada; 6 PBS Biotech Inc, 1183 Calle Suerte, Camarillo, CA 93012 USA

**Keywords:** Induced pluripotent stem cells, Vertical-wheel bioreactor, Computational fluid dynamics, Scale-up, Single cell, Harvest, Serial passage

## Abstract

**Background:**

Human induced pluripotent stem cells (hiPSCs) hold enormous promise in accelerating breakthroughs in understanding human development, drug screening, disease modeling, and cell and gene therapies. Their potential, however, has been bottlenecked in a mostly laboratory setting due to bioprocess challenges in the scale-up of large quantities of high-quality cells for clinical and manufacturing purposes. While several studies have investigated the production of hiPSCs in bioreactors, the use of conventional horizontal-impeller, paddle, and rocking-wave mixing mechanisms have demonstrated unfavorable hydrodynamic environments for hiPSC growth and quality maintenance. This study focused on using computational fluid dynamics (CFD) modeling to aid in characterizing and optimizing the use of vertical-wheel bioreactors for hiPSC production.

**Methods:**

The vertical-wheel bioreactor was modeled with CFD simulation software Fluent at agitation rates between 20 and 100 rpm. These models produced fluid flow patterns that mapped out a hydrodynamic environment to guide in the development of hiPSC inoculation and in-vessel aggregate dissociation protocols. The effect of single-cell inoculation on aggregate formation and growth was tested at select CFD-modeled agitation rates and feeding regimes in the vertical-wheel bioreactor. An in-vessel dissociation protocol was developed through the testing of various proteolytic enzymes and agitation exposure times.

**Results:**

CFD modeling demonstrated the unique flow pattern and homogeneous distribution of hydrodynamic forces produced in the vertical-wheel bioreactor, making it the opportune environment for systematic bioprocess optimization of hiPSC expansion. We developed a scalable, single-cell inoculation protocol for the culture of hiPSCs as aggregates in vertical-wheel bioreactors, achieving over 30-fold expansion in 6 days without sacrificing cell quality. We have also provided the first published protocol for in-vessel hiPSC aggregate dissociation, permitting the entire bioreactor volume to be harvested into single cells for serial passaging into larger scale reactors. Importantly, the cells harvested and re-inoculated into scaled-up vertical-wheel bioreactors not only maintained consistent growth kinetics, they maintained a normal karyotype and pluripotent characterization and function.

**Conclusions:**

Taken together, these protocols provide a feasible solution for the culture of high-quality hiPSCs at a clinical and manufacturing scale by overcoming some of the major documented bioprocess bottlenecks.

**Supplementary Information:**

The online version contains supplementary material available at 10.1186/s13287-020-02109-4.

## Introduction

Engineering approaches to understand and control stem cell behavior are needed to address major technology bottlenecks in bioprocessing knowledge [1–3]. Pluripotent stem cells have a high in vitro proliferation capacity and maintain the ability to differentiate into all three germ layers of the human body, making them an ideal cell platform for biomedical engineering applications [4–8]. With the ability to overcome ethical challenges associated with traditional embryonic cell sources, human induced pluripotent stem cells (hiPSCs) are of particular interest in research, clinical and manufacturing markets. Despite the demand for hiPSC production, a lack of standardized protocols and challenges with large-scale expansion to meet relevant cell quantities have prevented many advances in the field [9–11]. The number of high-quality cells required for treatment ranges from 10^9^ to 10^12^ depending on the therapeutic target, with therapeutic efficacy directly correlating to cell dose [12]. Bioreactors are the method of choice for controlled cell expansion, offering advantages including reduced labor and operating costs, greater cellular homogeneity, and more efficient cell expansion and differentiation capabilities compared to laboratory-scaled static culture flasks [13]. A drawback to the bioreactor environment is the potential for cells to be damaged by high shear stress which can tear apart cells and cell aggregates, resulting in lower cell quality and cell yield [14]. While several studies have investigated the production of hiPSCs in bioreactors, the use of conventional horizontal-impeller, paddle, and rocking-wave mixing mechanisms has demonstrated unfavorable hydrodynamic environments for hiPSC growth and quality maintenance. Current studies achieve only moderate cell fold increases during the expansion phase, and there exists a lack of scalable protocols for the inoculation and harvesting phases [15–21].

A major hurdle for hiPSC expansion in bioreactors has been defining a scalable cell inoculation protocol that successfully maintains cell growth rates without sacrificing cell quality. Single-cell enzymatic dissociation of hPSCs has been reported to result in a drastic loss of cell viability. Thus, hPSCs are generally plated as clumps and grown as colonies or aggregates [11]. Even with a static growth platform, non-colony-type monolayers have resulted in low cell production and reported chromosomal abnormalities and potential selection pressure for mutated cells [22, 23]. While clump seeding has been widely reported as an inoculation strategy for bioreactor culture [24–30], it produces a bottleneck in scalability. It is also difficult to control the clump size, resulting in heterogeneity of bioreactor aggregates leading to increased apoptosis and spontaneous differentiation [11, 24]. To avoid the formation of large aggregates prone to necrotic centers, optimal cell inoculation methods and passaging schedules should be determined. Single-cell inoculation methods have been tested with traditional bioreactor models; however, studies have required large cell seeding densities which resulted in low cell production [11].

Downstream hiPSC bioreactor operations also lack scalable protocols. Harvesting is a critical step in serial passaging and recovery of the final cell product [9], but excessive shear during the harvesting process can alter cell phenotype [31]. Few studies have investigated potential methods for full bioreactor harvesting of hiPSC aggregate culture. Publications only collect small (1–5 mL) aggregate samples from the bioreactor to dissociate for cell counts using enzymatic and mechanical dissociation techniques which cannot be translated to harvesting [9, 10, 32].

In this study, we utilized computational fluid dynamics (CFD) modeling to map out the hydrodynamic environment of a vertical-wheel bioreactor produced by PBS Biotech, Ltd. Hydrodynamic forces are difficult to study empirically due to confounding dynamic variables present at any given instant [33]. CFD modeling can be applied to understand hydrodynamics in stirred suspension bioreactors. This in turn affects cell viability, proliferation, pluripotency, and differentiation. The CFD model allowed us to confirm that the vertical-wheel platform uniquely combines radial and axial flow components producing more uniform distributions of hydrodynamic forces and better scalability compared to traditional bioreactor geometries [34, 35]. We hypothesized that the hydrodynamic environment of the vertical-wheel bioreactor would make it the ideal platform for protocol development to overcome challenges in hiPSC bioprocess scale-up. Table 1 highlights the major geometrical differences and known advantages of the proposed vertical-wheel bioreactor compared to traditional horizontal-blade bioreactors [36]. By adjusting the vertical-wheel operating parameters, we showed success in single-cell hiPSC inoculation resulting in over 30-fold expansion in 6 days. This could not be mimicked in traditional horizontal-blade bioreactors which produced heterogenous cell aggregates and minimal cell expansion when seeded as single cells. Using enzymatic dissociation within the agitated vertical-wheel bioreactor, we designed a harvesting protocol capable of achieving a recovery efficiency of over 95%. The optimized inoculation and harvesting protocols were combined in a serial passage and scale-up study that showed reproducible hiPSC growth that maintains a normal karyotype, a positive expression of pluripotency markers, and the functional ability to differentiate into all three germ layers.

**Table 1:**
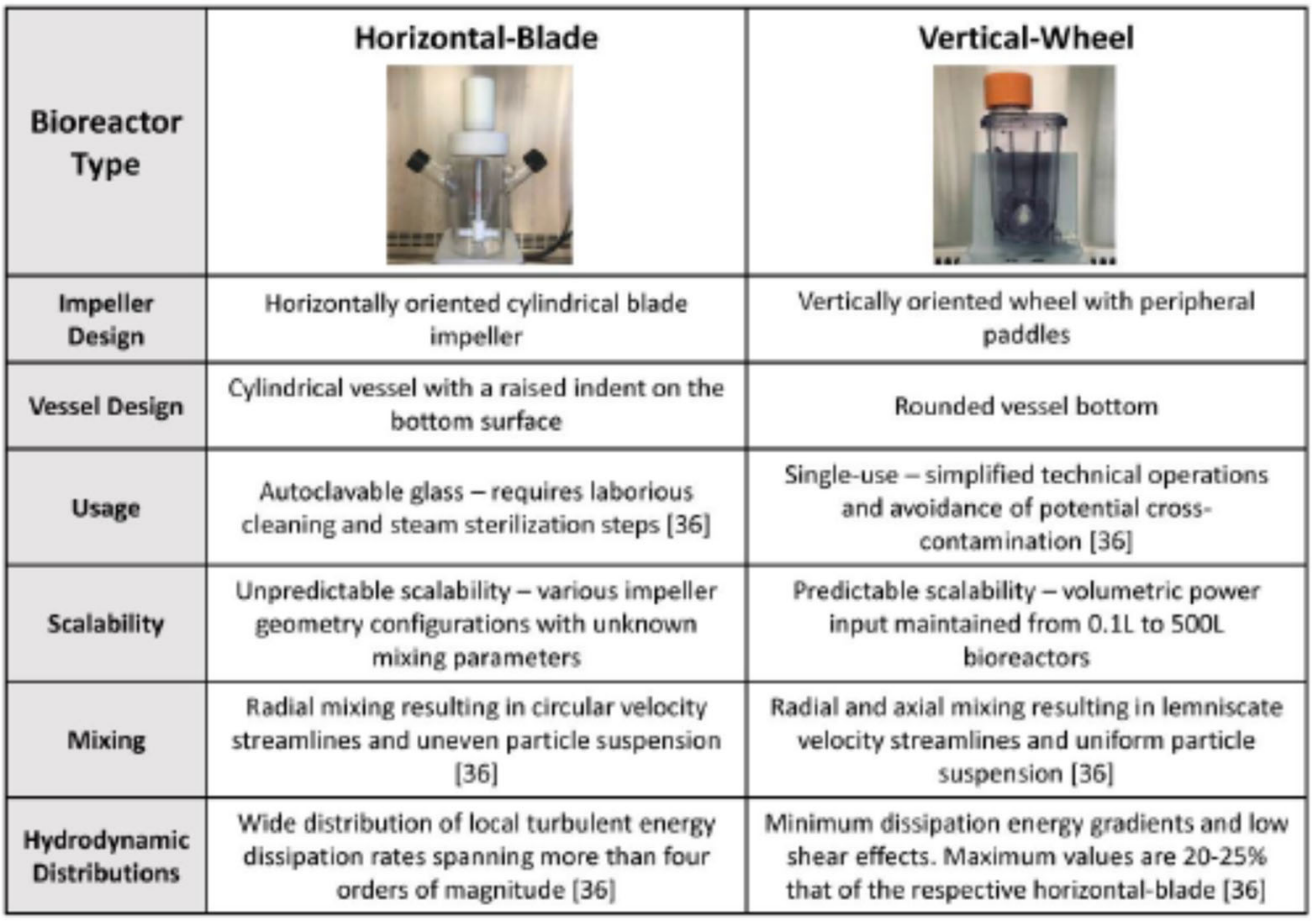
The major geometrical differences and known advantages of the proposed vertical-wheel bioreactor compared to traditional horizontal-blade bioreactors

## Materials and methods

### Computational fluid dynamics modeling

The 100-mL vertical-wheel bioreactor (PBS Biotech, Camarillo, USA) was modeled with CFD simulation software Fluent 16.2 (ANSYS Inc. Cannonsburg, USA). Fluent employs a finite volume formulation to numerically solve CFD models. Virtual geometry models of the reactor were created using the computer-aided design (CAD) software AutoCAD and imported into the meshing software ICEM ANSYS. The reactor geometry was discretized using tetrahedral elements and boundary conditions were prescribed at different surfaces. Wall boundary conditions were applied to the vessel wall and impeller, indicating regions of zero normal velocity and no tangential velocity relative to the wall (no slip condition). The liquid surface was modeled with a free surface boundary condition, defining a fixed, frictionless wall with no tangential velocity restrictions, and zero normal velocity. The impeller rotation was implemented using a moving reference frame with an interface boundary condition used between the rotating and stationary domains.

A semi-implicit method for pressure-linked equations (SIMPLE) algorithm was used to numerically solve the realizable k-epsilon Navier-Stokes equations. The k-epsilon model is one of the most widely used turbulent models for simulating the hydrodynamic environment in suspension bioreactors [37–39] and has been validated with particle image velocimetry (PIV) [40]. Navier-Stokes utilizes Eqs. 1 and 2 to represent the transport of mass and momentum through viscous fluid:

1 $$ \frac{\partial \rho }{\partial t}+\nabla \bullet \left(\rho u\right)=0 $$

2 $$ \frac{\partial \rho u}{\partial t}+\nabla \left(\rho u\bigotimes u\right)=-\nabla P+\mu {\nabla}^2u+\rho g $$

In the above equations, *ρ* is the density, *u* is the Cartesian velocity vector, *t* is time, *P* is pressure, *μ* is viscosity, and *g* is the gravity vector. Water at 37 °C with a density of 0.993 g/cm^3^, a dynamic viscosity of 7.01 × 10^−4^ kg/(m s), and kinematic viscosity of 0.696 mm^2^/s was used to simulate the fluid inside the reactor. In order to represent turbulence in the system, the realizable k-epsilon model implements two additional transport equations to account for kinetic energy and energy dissipation rate.

All equations were discretized using a second-order upwind scheme. Models were generated at agitation rates of 20, 40, 60, 80, and 100 rpm, each run for a flow time of 5 s with time steps chosen to ensure the Courant-Friedrich-Lewy (CFL) number remained below 1. This guaranteed that the fluid element would cross from one end of the mesh element to the other in a single time step. Post processing was performed on each simulated model to derive velocity, shear stress (force acting on a surface parallel to the plane in which it lies), and energy dissipation rate (energy lost by viscous forces) distributions.

### Static culture of hiPSCs

hiPSC line 4YA, passage numbers 40 to 45, were used for all experiments in this study. These cells were obtained from Dr. James Ellis’ laboratory at the University of Toronto (Toronto, Canada). For expansion prior to inoculation in bioreactor culture, hiPSCs were grown in T-75 flasks (Cat#156599, Thermo Scientific) maintained under standard culture conditions (37 °C and 5% CO_2_). Flasks were coated with feeder-free substrate hESC-qualified Matrigel (Cat#354277, Corning Life Sciences) in DMEM/Hams F-12 (Cat#10-090-CV, Corning Life Sciences) for 2 h at room temperature. The cells were inoculated into T-75 flasks at a density of 15,000 cells/cm^2^ with 15 mL/flask mTeSR1 medium (Cat#85851, STEMCELL Technologies) supplemented with 10 μM Y-27632 (Cat#72304, STEMCELL Technologies). Daily medium replacements were carried out, excluding the addition of Y-27632. When approximately 80% confluency was reached (3–4 days), hiPSCs from static were passaged. Static cultures were washed once with Ca^2+^- and Mg^2+^-free phosphate buffer solution (PBS) and treated with 5 mL/flask 0.5 mM EDTA·4Na Accutase (Cat#07920, STEMCELL Technologies) supplemented with 10 μM Y-27632 and incubated at 37 °C for 5 min. Medium supplemented with 10 μM Y-27632 was added at a 1:1 ratio of Accutase to dilute the enzyme. The culture was then transferred to a conical tube to be centrifuged at 300*g* for 5 min. The supernatant was discarded, and the cell pellet was resuspended in a medium supplemented with 10 μM Y-27632 to be counted and inoculated into bioreactor culture.

### Suspension culture of hiPSCs

This study used 100-mL working volume horizontal-blade, glass bioreactors (Corning Style Spinner Flask, NDS Technologies Inc.) and 100-mL and 500-mL working volume single-use, vertical-wheel bioreactors (PBS Biotech Ltd). Constant mixing was maintained at agitations rates of 40, 60, and 80 rpm in standard culture conditions of 37 °C and 5% CO_2_. hiPSCs were inoculated at a density of 20,000 cells/mL and cultured at maximum working volumes (100 mL and 500 mL) of mTeSR1 medium supplemented with 10 μM Y-27632. Experiments utilized either single-cell or pre-formed aggregate inoculation methods. Bioprocess parameters, including the selected low cell seeding density and protocol used to pre-form aggregated cell clumps, were adopted from a previous study as a positive control to compare single-cell inoculation in the vertical-wheel bioreactor [41]. When inoculated as single cells, hiPSCs removed from static culture were counted and added directly into the agitated bioreactor culture. When inoculated as pre-formed aggregates, hiPSCs removed from static culture were counted and inoculated into 6-well suspension culture plates (Cat#657-185, Greiner CELLSTAR) at a concentration of 200,000 cells/cm^2^ in 2.0 mL/well mTeSR1 supplemented with Y-27632. Wells were left in the incubator for 4 h to pre-form aggregated cell clumps which were then inoculated into the bioreactor.

Experiments studying the effects of nutrient availability in 100 mL and 500 mL fed-batch conditions involved a 50% mTeSR1 medium exchange (excluding Y-27632) on day 4 of culture. To perform the medium exchange, bioreactors were brought under a laminar flow hood where cell aggregates settled for 5 min. Media were aspirated from the top surface of the bioreactor and added to a conical tube to be centrifuged at 300*g* for 3 min. Media from the conical tube were discarded, and the remaining cell pellet was resuspended in fresh mTeSR1 media to be added back into the bioreactor.

### Cell counts and aggregate sizing

To study the growth kinetics of hiPSCs in bioreactor culture, daily samples were taken with cell counts performed in duplicates. Samples of 1.0–5.0 mL were removed using a serological pipette during bioreactor agitation to minimize settling of the aggregates. The samples were centrifuged at 300*g* for 5 min. The supernatant was discarded, and the cell pellet was resuspended in 1.0 mL of Accutase and left in a 37 °C water bath for 5–7 min. The cell solution was then gently pipetted 3 times followed by the addition of 1.0 mL of the medium used to dilute the enzyme. The sample was centrifuged at 500*g* for 5 min, the supernatant was discarded, and the cell pellet was resuspended in 0.5–1.0 mL medium. Two, 200 μL aliquots were taken from each cell sample for viable cell counts using the NucleoCounter NC-200 (ChemoMetec, Denmark), an automated cassette counter which analyzes samples stained with fluorescent dyes Acridine Orange and DAPI. These counts were used to generate cell growth curves and calculate fold expansion utilizing Eq. 3:

3 $$ \mathrm{Fold}\ \mathrm{expansion}=\frac{X_2}{X_1} $$

where *X*_1_ and *X*_2_ are the viable cell densities (cells/mL) at the beginning and end of the culture passage.

To determine average aggregate size and size distributions, 1.5-mL samples were removed using a serological pipette from the bioreactors and added into 12-well plates for visualization. Images were taken using a Zeiss Axiovert 25 microscope (Carl Zeiss) with AxioVision software used for measurements. Aggregates were defined as multi-cellular spheroids with a diameter greater than 50 μm. The diameter for each aggregate was determined by taking the average of the greatest length across the aggregate and the length perpendicular to the greatest length. A minimum of 100 aggregates were sized per condition.

### Bioreactor harvesting

An in-vessel harvest protocol was developed through the testing of various proteolytic enzymes and agitation exposure times. Proteolytic enzymes tested were Accutase (Cat#07922, STEMCELL Technologies), TrypLE (Cat# 12605028, ThermoFisher), and 0.05% Trypsin-EDTA (Cat#25300062, ThermoFisher). The bioreactor was brought under the laminar flow hood where aggregates settled for 5 min. Media from the bioreactor were aspirated into conical tubes, leaving approximately 3 mL of aggregate culture in the bioreactor. Conical tubes were centrifuged at 300*g* for 5 min. Media from the conical tubes were discarded, and the remaining cell pellets were resuspended in 20 mL of proteolytic enzyme with 10 μM Y-27632 and added back into the bioreactor aggregate culture. The bioreactor was then placed back in the incubator (37 °C, 5% CO_2_) and agitated at 80 rpm. Every 5 min, for a total of 30 min, the bioreactor was quickly brought under the laminar flow hood where 200-μL samples were removed for cell counts and phase-contrast imaging. Cell counts were performed using the NucleoCounter NC-200 to obtain a viable cell density and a percent of cells in aggregates (defined by the instrument as a cluster of 5 or more cells). To calculate the dissociation efficiency at each time point, Eq. 4 was used:

4 $$ \mathrm{Dissociation}\ \mathrm{Efficiency}=\frac{A}{B}\times 100-\mathrm{cells}\ \mathrm{in}\ \mathrm{aggregates} $$

where variables *A* and *B* are total cell numbers in the reactor before and after the harvesting procedure. The total cells in the reactor prior to harvesting were calculated through Eq. 5:

5 $$ A=\mathrm{Ave}\frac{\mathrm{cell}}{\mathrm{mL}}\ \mathrm{sample}\ \mathrm{count}\times \mathrm{reactor}\ \mathrm{volume}\ \mathrm{predissociation} $$

where the average sample count was determined through bioreactor cell samples (1.0 mL) dissociated via traditionally counting methods described above. The reactor volume predissociation was the sum of the media transferred to conical tubes and the remaining aggregate culture in the bioreactor (approximately 3 mL). Total cells in the reactor after the harvesting procedure was calculated through Eq. 6:

6 $$ B=\mathrm{Ave}\frac{\mathrm{cell}}{\mathrm{mL}}\mathrm{sample}\ \mathrm{count}\times \mathrm{reactor}\ \mathrm{volume}\ \mathrm{post}\ \mathrm{dissociation} $$

where the average sample count was determined from the 200-μL samples taken directly from the bioreactor during the harvesting procedure. The reactor volume post dissociation was the sum of the proteolytic enzyme volume added to the bioreactor (20 mL) and the remaining aggregate culture in the bioreactor prior to harvesting (approximately 3 mL).

### Karyotyping

Samples of bioreactor-generated aggregates were incubated in 10-mL bioreactors in medium supplemented with 0.1 μg/mL KaryoMax Colcemid (Cat#15212012, ThermoScientific) for 4 h. The aggregates were then enzymatically dissociated as previously described. Single cells were collected by centrifugation, suspended in 0.075 M KCl hypotonic solution (Cat#P217-500, Fischer Scientific), and incubated at 37 °C for 25 min. Cells were then fixed with 3:1 methanol to acetic acid solution (Cat#A412-4, Fisher Scientific, Cat#AX0073, EMD), and chromosome preparations were GTG-banded using standard cytogenetic techniques. Karyograms were analyzed according to the ISCN standards at ~ 450 band resolution using the Ikaros karyotyping system (Metasystems).

### Aggregate immunocytochemistry

Aggregate samples containing 1E6 cells were removed from the bioreactor culture and added into microcentrifuge tubes (Cat#10011-724, VWR). Aggregates were rinsed twice with PBS and resuspended in 0.5 mL of fixation buffer (Cat#FC001, R&D Systems) to be incubated for 1 h at room temperature. Aggregate samples were then rinsed twice with PBS and resuspended in 200-μL permeabilization buffer (Cat#FC005, R&D Systems) with 1 μg/10^6^ cells antibody stain and 1 μM/10^6^ cells nuclei stain and incubated for 3 h at room temperature. Conjugated antibody stains for SSEA-4 (Cat#FAB1435F, R&D Systems), TRA-1-60 (Cat# FAB4770P, R&D Systems), and Nanog (Cat#MABD24A4, Millipore Sigma) were used along with the nuclei stain To-Pro-3 Iodide Nucleic Acid Stain (Cat#T3605, Thermo Fisher). Cells were then rinsed twice with PBS and imaged using a Carl Zeiss Laser Scanning Microscope 700 with lasers at 488 nm and 639 nm and corresponding filter sets.

### Tri-lineage differentiation

Bioreactor-cultured hiPSC aggregates were differentiated into cardiomyocytes, hepatocytes, and neural rosettes using the previously published protocols [42–44]. The aggregates were collected on day 12 post-inoculation and were either dissociated into single cells as described in the “Materials and methods” section or directly plated as aggregates for in vitro differentiation. For both cardiomyocyte and hepatocyte differentiation, hiPSCs were seeded at a density of 2E5 cells/plate on Matrigel-coated FluoroDish Cell Culture Dish plates (Cat#FD35-100, World Precision Instrument) containing mTeSR1 supplemented with Y-27632 and cultured to 80–90% confluency before undergoing differentiation [42, 43]. Mature cardiomyocytes and hepatocytes at day 20 of differentiation were used for the analysis. For neural rosette differentiation, hiPSC aggregates were cultured on poly-l-ornithine (Cat#A-004-C, Sigma Aldrich)-coated FluoroDish cell culture dish plates in DMEM/F12 medium supplemented with 5% KnockOut serum replacement (Cat# 10828010, Thermo Fisher Scientific), 0.1 mM non-essential amino acids (Cat# 11140050, Thermo Fisher Scientific), 0.1 mM 2-mercaptoethanol (Cat#21985023, Thermo Fisher Scientific), and 1% penicillin-streptomycin (Cat#15140122, Thermo Fisher Scientific) for 4 days. Further, the aggregates were transferred onto Matrigel-coated plates in Neurobasal™ Medium (Cat#21103049, Thermo Fisher Scientific) supplemented with B27 without retinoic acid (Cat#12587010, Thermo Fisher Scientific), N2 supplement (Cat#17502048, Thermo Fisher Scientific), 0.005% bovine serum albumin (Cat#15260037, Thermo Fisher Scientific), and 1 mM sodium pyruvate (Cat#11360-070, Thermo Fisher Scientific) for an additional 5 days before analysis. Differentiated cardiomyocytes and hepatocytes were analyzed by whole-mount immunostaining and confocal imaging using Cardiac Troponin T antibody (Cat#MA5-12960, 5 μg/mL, Thermo Fisher Scientific), HNF-4-alpha antibody (Cat#ab92378, 1:100, Abcam), and CYP3A4 antibody (Cat# MA5-17064, 1:200, Thermo Fisher Scientific). Neural rosettes were analyzed using Pax-6 (Cat# PRB-278P, 1:100, BioLegend) and Tubulin β 3 (TUBB3) (Cat#801202, 1 μg/mL, BioLegend) antibodies.

### RNA isolation and reverse transcription (RT) quantitative (q) polymerase chain reaction (PCR) (RT-qPCR)

hiPSC aggregates were collected on day 6 and day 12 post-inoculation and used for RNA isolation. Total RNA was extracted using PureLink™ RNA Mini Kit (Cat#12183018A, Thermo Fisher Scientific) according to the manufacturer protocol, followed by DNAse I digestion using DNAse I Amplification Grade (Cat#18068015, Thermo Fisher Scientific). Next, 500 ng RNA was used for cDNA synthesis using Superscript® IV Reverse Transcriptase (Cat#18090010, Thermo Fisher Scientific) and 50 μM Oligo (dT)20 Primer (Cat#18418020, Thermo Fisher Scientific) according to the manufacturer’s instructions. To quantitate transcripts, the subsequent RT-qPCR gene expression analysis was performed on Applied Biosystems (Thermo Fisher Scientific) using Fast SYBR™ Green Master Mix (Cat#4385612, Thermo Fisher Scientific). For each sample, relative mRNA expression was quantified relative to the housekeeping gene GAPDH and was normalized to static cultured hiPSC level (= 1). The relative quantification (RQ) was completed based on comparative C_T_ (ΔΔC_T_) through the 2^–ΔΔCT^ method. The gene expression results are shown as relative mRNA expression (RQ) to static cultured hiPSCs (RQ = 1). At least two technical and three biological replicates were assayed for all quantitative RT-PCR reactions. Pluripotency-associated genes, Oct-4, Sox2, Nanog, Klf4, and Rex1, were used for RT-qPCR. The primer sequences for SYBR Green probe are listed in Supplementary Table 1.

### Statistics

Statistical analysis was done using GraphPad Prism (v6.0). A one-way ANOVA followed by Dunnett multiple comparison test was used for all growth curve and aggregate size comparisons. Cell samples were collected from *n* = 4 stirred suspension bioreactors at each condition. The *P* values were set at 0.05, and all graphs are presented with a ± standard error of the mean (SEM). A one-way analysis of variance (ANOVA) followed by Tukey’s multiple comparison test was used for RT-qPCR for statistical analysis. The significance was set at *P* < 0.05 using GraphPad Prism.

## Results

### Vertical-wheel bioreactor CFD modeling

**Fig. 1:**
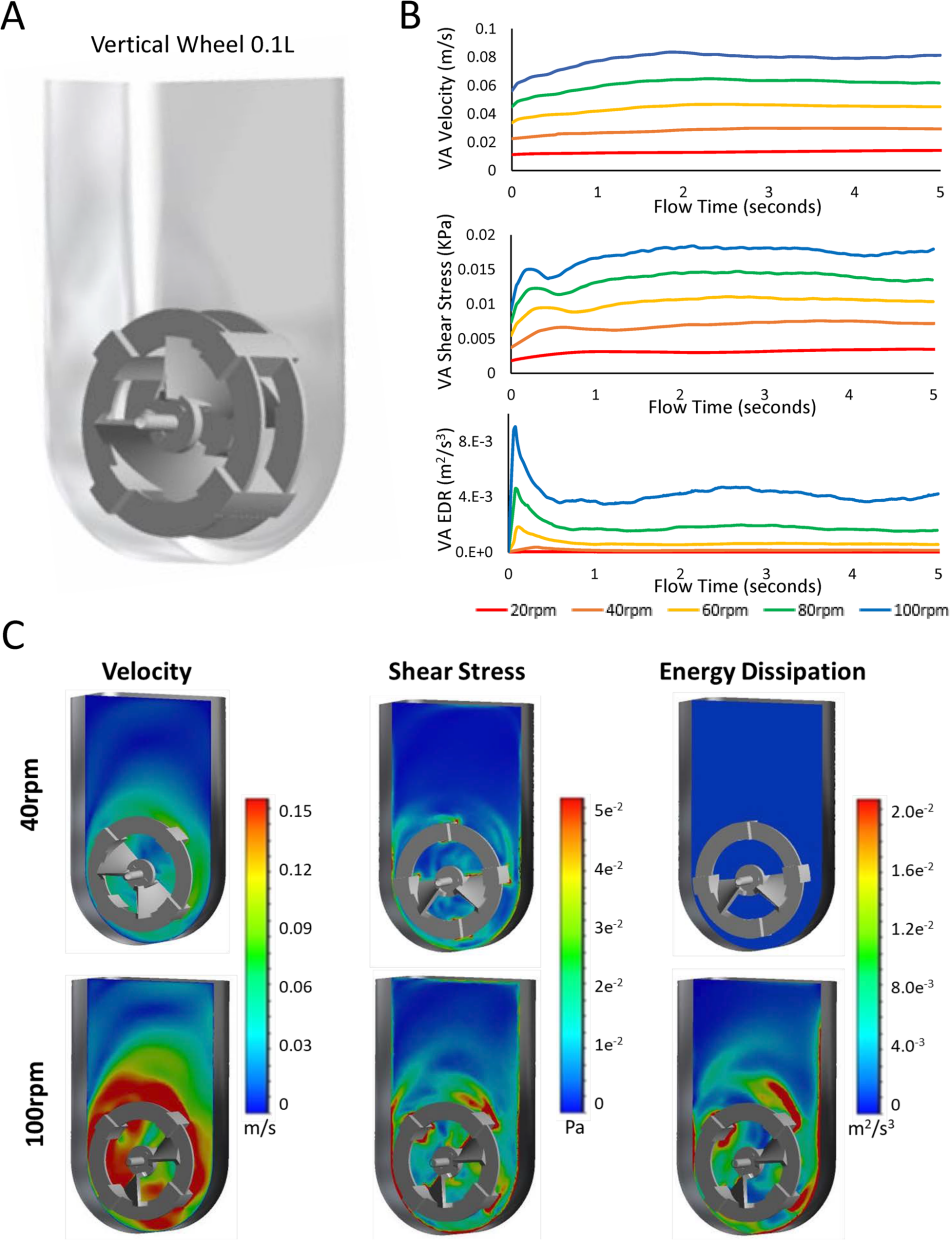
**a** Geometric outline of the 0.1 L PBS vertical-wheel bioreactor input into Ansys Fluent CFD modeling software. **b** Computational values for the volume average (VA) velocity, shear stress, and energy dissipation rate run for a flow time of 5 s at agitation rates between 20 and 100 rpm. **c** Vertical heatmap slices highlighting distributed areas of relatively high (red) and relatively low (blue) areas of local velocity, shear stress, and energy dissipation rate

**Fig. 2:**
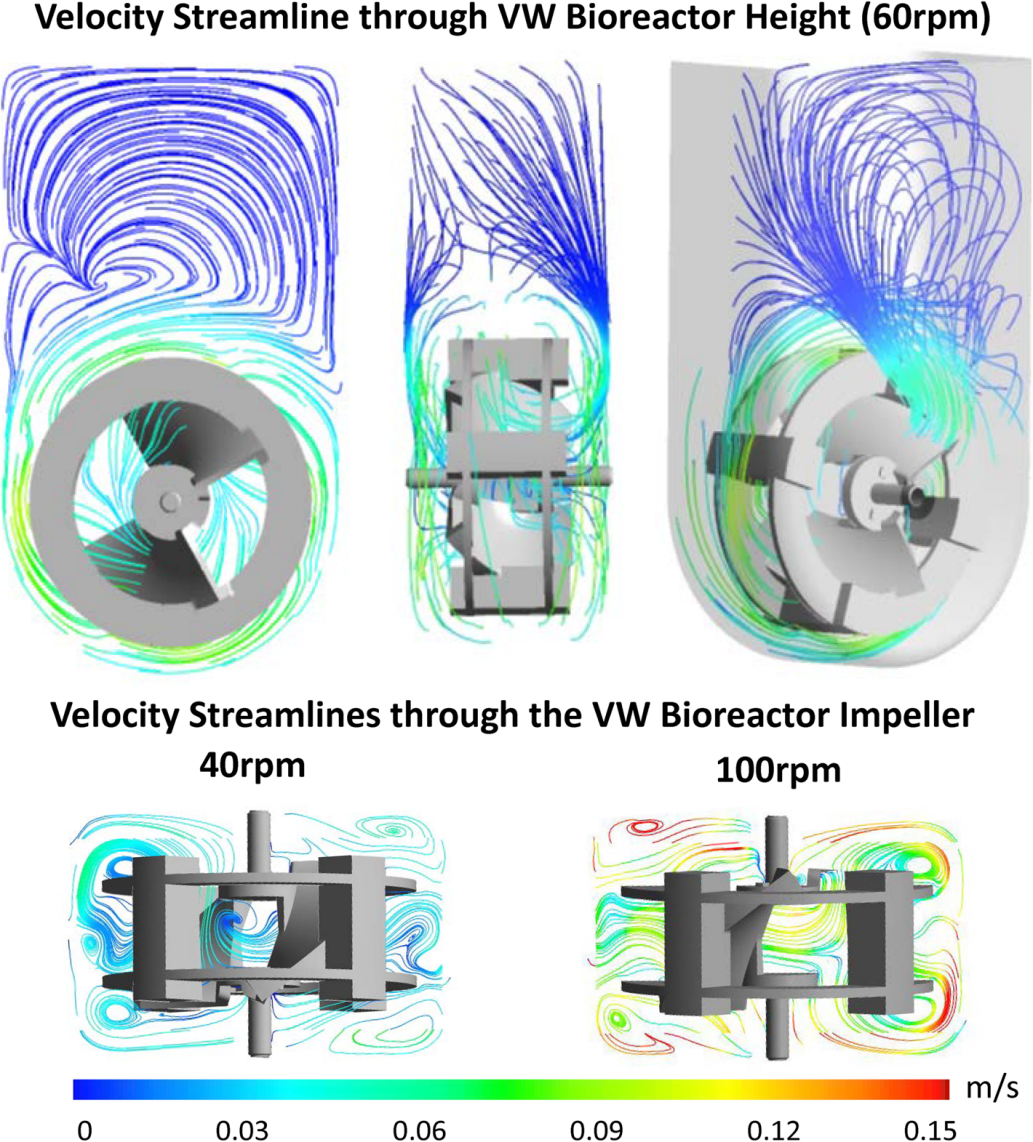
Velocity flow patterns within the 0.1-L vertical-wheel bioreactor generated through computational fluid dynamics modeling. ANSYS CFD-Post software was used to generate the velocity streamlines with results taken at the final simulation time step (5 s of flow time). The velocity color scale was set to a minimum of 0 m/s (blue) and maximum of 0.15 m/s (red)

To investigate the hydrodynamic environment, the vertical-wheel bioreactor, geometrically outlined in Fig. 1a, was modeled at agitation rates between 20 and 100 rpm. Figure 1b displays the change in volume average hydrodynamic variables (velocity, shear stress, and energy dissipation rate) from when the model was initiated through 5 s of flow time. Steady state is reached between 2 and 3 s for all tested agitations, evident by the plateau in measured values. The exception being 100 rpm, where the volume average energy dissipation rate continues to fluctuate between 3.7E−3 m^2^/s^3^ and 4.7E−3 m^2^/s^3^ due to an increase in turbulent energy. Both volume average velocity and volume average shear stress increase in a linear fashion with respect to agitation rate. Conversely, volume average energy dissipation rate increases exponentially with respect to agitation rate. It is evident from the vertical slices in the bioreactor (Fig. 1c) that at a lower operating condition, 40 rpm, hydrodynamic forces within the reactor are very consistent. The distribution in energy dissipation rate throughout the reactor height is particularly narrow, with no noticeable changes within the rotating domain at the given colorimetric scale. At a higher operating condition, 100 rpm, hydrodynamic forces show an increase in variability within the reactor volume, with much greater forces acting around the impeller blades. The difference in maximum force values, which are often calculated to keep constant in scale-up, are orders of magnitude higher than the volume average values. At 100 rpm, for instance, the maximum energy dissipation rate is 8.9E−1 m^2^/s^3^ whereas the volume average energy dissipation rate is 4.2E−3 m^2^/s^3^. What is particularly interesting is the CFD generated flow patterns within the vertical-wheel bioreactor, shown in Fig. 2. The velocity streamlines in the vertical-wheel bioreactor display a lemniscate (figure-eight) profile. Unlike traditional horizontal-impeller bioreactors, the fluid in the vertical-wheel reactor moves throughout the entire liquid width and height. The fluid streamlines weave between the pitched wheel blades with increased velocity corresponding to an increase in agitation.

### Single-cell bioreactor inoculation

**Fig. 3:**
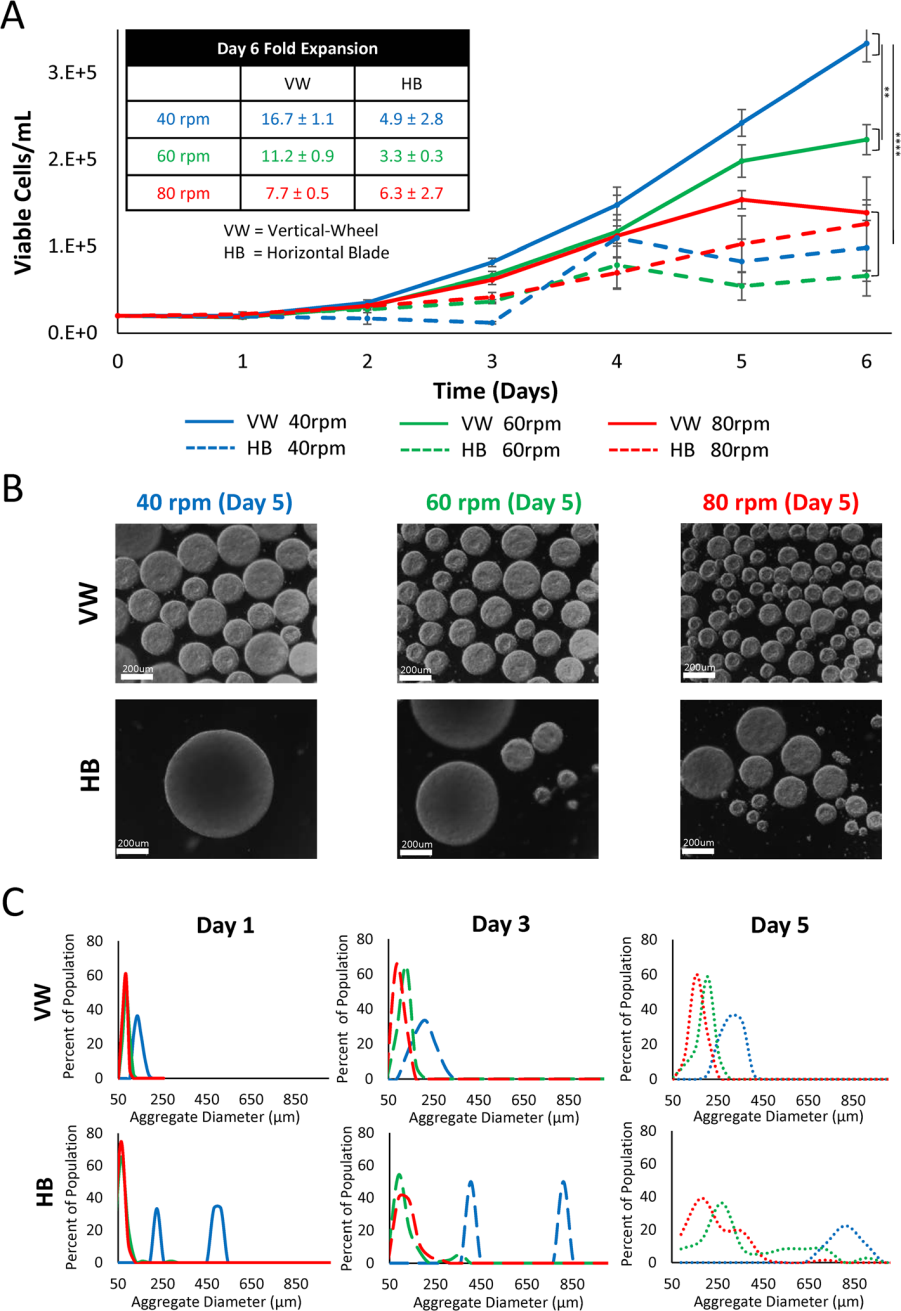
**a** Growth kinetics, **b** representative brightfield microscopic images, and **c** aggregate size distributions of hiPSCs seeded as single cells and cultured in either vertical-wheel or horizontal-blade bioreactors at agitation rates of 40 rpm, 60 rpm, and 80 rpm with no medium exchange (batch) for 6 days (scale bar = 200 μm). Cell samples were collected from *n* = 4 stirred suspension bioreactors at each condition. The *P* values were set at 0.05 and all graphs are presented with a ± standard error of the mean (SEM)

To first investigate the potential of single-cell inoculation, hiPSCs were seeded into 100-mL working volume horizontal-blade and vertical-wheel bioreactors operated at 40 rpm, 60 rpm, and 80 rpm for 6 days of batch culture. As is evident from the growth curve and fold-expansion data (Fig. 3a), single-cell inoculation of hiPSCs in the vertical-wheel reactor was quite successful, reaching a maximum expansion of 16.7 ± 1.1-fold at 40 rpm. In contrast, fold expansion in the horizontal-blade bioreactor was minimal, reaching a maximum of 6.3 ± 2.7-fold at 80 rpm. The reduced cell yield in the horizontal-blade reactor is likely linked to poor mixing that results in large, heterogeneous aggregates pictured in Fig. 3b. The aggregate distribution graphs for the horizontal-blade reactor (Fig. 3c) acquire either bi-model peaks or large, flat distributed averages, indicative of unhealthy aggregate morphology. By day 5 of culture, hiPSC aggregates in the horizontal-blade reactors reached over 400 μm in diameter. Beyond this aggregate size threshold, necrosis is expected to occur with low levels of oxygen and nutrients diffusing into the center of the aggregate, resulting in heterogeneity in cell growth and differentiation potential [45]. hiPSCs cultured in the vertical-wheel bioreactors maintained consistent aggregate sizes with a single, narrow peak distribution at all tested agitation rates. Day 5 average aggregate sizes in the vertical-wheel reactor seeded with single cells remained below the threshold aggregate size, ranging between 169.4 μm ± 5.5 μm and 275.45 μm ± 6.9 μm in diameter at 80 rpm and 40 rpm respectively.

**Fig. 4:**
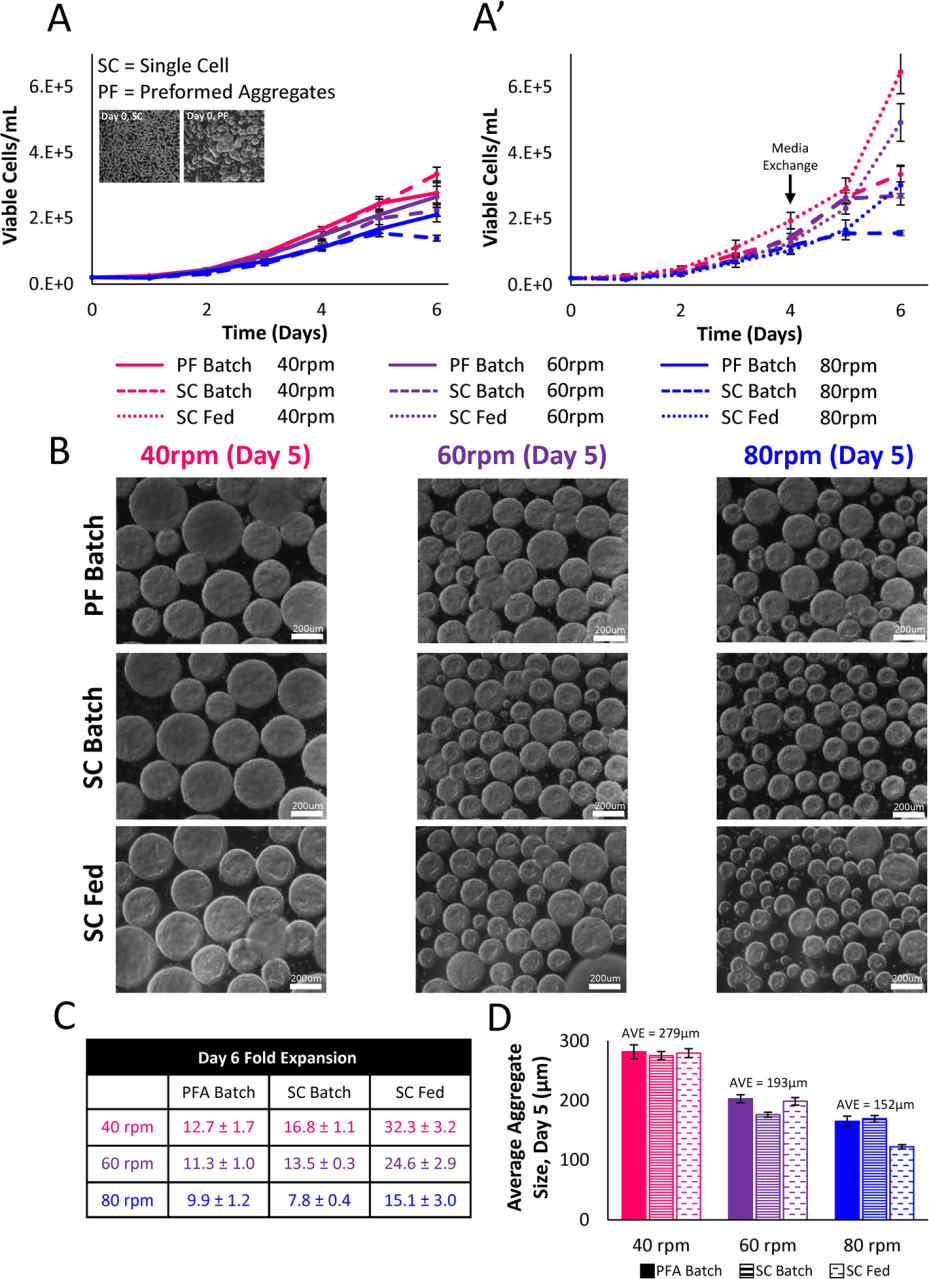
**a** Growth kinetics for hiPSCs seeded as either single cells or pre-formed aggregates cultured in vertical-wheel bioreactors at agitation rates of 40 rpm, 60 rpm, and 80 rpm with no medium exchange (batch) for a period of 6 days. **a**’ Growth kinetics for hiPSCs seeded as single cells in vertical-wheel bioreactors at agitation rates of 40 rpm, 60 rpm, and 80 rpm with either no medium exchange (batch) for 6 days or a 50% medium exchange on day 4 (fed) of the 6-day culture period. **b** Representative brightfield microscopic images, **c** day-6 fold expansions, and **d** average aggregate sizes of hiPSCs cultured in vertical-wheel bioreactors under pre-formed (PF) batch, single-cell (SC) batch, and single-cell (SC) fed conditions at agitation rates of 40 rpm, 60 rpm, and 80 rpm (scale bar = 200 μm). Cell samples were collected from *n* = 4 stirred suspension bioreactors at each condition. The *P* values were set at 0.05 and all graphs are presented with a ± standard error of the mean (SEM)

Next, pre-formed aggregate inoculation and single-cell inoculation methods were compared in the vertical-wheel bioreactors. Under batch culture conditions, there were no differences in growth at corresponding agitation rates (Fig. 4a), with aggregate morphology remaining consistent between inoculation methods (Fig. 4b). At the tested agitation rates and inoculation methods, the cells experienced a lag phase on day 1 and entered the exponential growth phase on day 2 of culture. Batch and fed-batch single-cell inoculation in the vertical-wheel bioreactors was then compared (Fig. 4a’). At all tested agitation rates, fed-batch culture resulted in maximum fold expansions that were approximately twice that of batch culture conditions (Fig. 4c). Final cell concentrations at 40 rpm were significantly higher than those at 60 rpm and 80 rpm, with a maximum expansion of 32.3 ± 3.2-fold reached on day 6 of fed-batch culture. While there were significant differences in average aggregate size between the tested agitation rates, with average aggregate size decreasing in correspondence to an increase in agitation rate, average aggregate size within each agitation rate seeded as pre-formed or single cells remained the same (Fig. 4d).

### Bioreactor harvesting

**Fig. 5:**
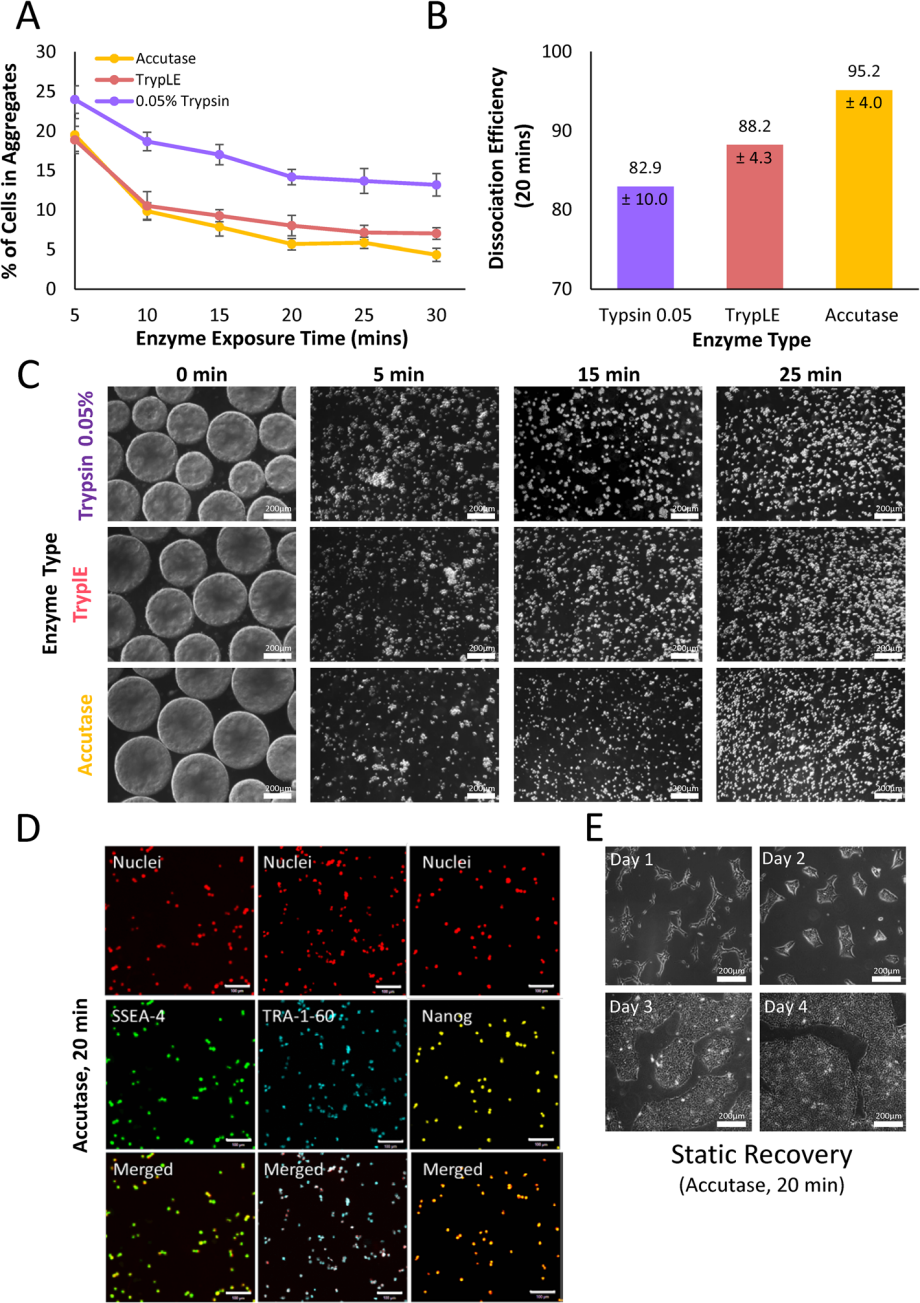
**a** Percent of cells in aggregates, **b** dissociation efficiency, and **c** representative brightfield microscopic images of hiPSC aggregates exposed to either Accutase, TrypLE, or 0.05% Trypsin for periods of 5–30 min during full bioreactor harvesting (scale bar = 200 μm). **d** Representative confocal microscope images of hiPSC single-cell samples taken from the full bioreactor harvest (Accutase for 20 min) and stained for pluripotency markers SSEA-4, TRA-1-60, and Nanog (scale bar = 100 μm). **e** Representative brightfield microscope images of hiPSCs taken from the full bioreactor harvest (Accutase for 20 min) and seeded onto Matrigel-coated dishes for static recovery (scale bar = 200 μm). Cell samples were collected from *n* = 4 stirred suspension bioreactors at each condition. The *P* values were set at 0.05 and all graphs are presented with a ± standard error of the mean (SEM)

An optimized in-vessel harvesting protocol was developed by studying the effects of enzyme type and agitation exposure time on the dissociation efficiency and percentage of cells in aggregates. Accutase, TrypLE, and 0.05% Trypsin-EDTA were tested at exposure times of 5, 10, 15, 20, 25, and 30 min. A large reduction in the percent of aggregates occurred between 5 and 10 min for all tested enzymes (Fig. 5a). A further decrease in the number of aggregates continued until 20 min for all tested enzymes. After 20 min, the percent of aggregates remains plateaued, indicating no further dissociation activity was occurring. When comparing enzyme types, the percent of aggregates remaining and the dissociation efficiency at the end of 20 min were considered. The dissociation efficiency at 20 min was calculated by comparing cell sample counts prior to the full reactor harvest with samples taken during the harvest and factoring in the percent of cells that remained in aggregates. Of the tested enzymes, 0.05% Trypsin resulted in the poorest performance, with a calculated dissociation efficiency of 82.9% ± 10.0%. The use of Accutase resulted in the highest dissociation efficiency of 95.2% ± 4.0% and was therefore selected as the optimal enzyme for the in-vessel harvest protocol (Fig. 5b).

Figure 5c provides a visualization to support this observation where images were taken before dissociation began and at 5, 15, and 25 min. After only 5 min, dissociation was prevalent, with all aggregates visibly breaking apart into small clusters or single cells. By 15 min, most of the smaller aggregates had dissociated, and by 25 min, mostly single cells remained. As no further dissociation occurred after 20 min, it was selected as the optimal agitated exposure time.

Following the bioreactor harvest using Accutase for 20 min, single-cell samples were assessed using immunocytochemistry. The cells maintained positive expression for human pluripotency markers SSEA-4, TRA-1-60, and Nanog (Fig. 5d). In addition, proliferative capabilities were assessed by recovering the dissociated single cells in static T-75 flasks coated with Matrigel. Typical hiPSC static morphology and growth was observed over 4 days of recovery culture (Fig. 5e).

### Serial passaging and quality testing

**Fig. 6:**
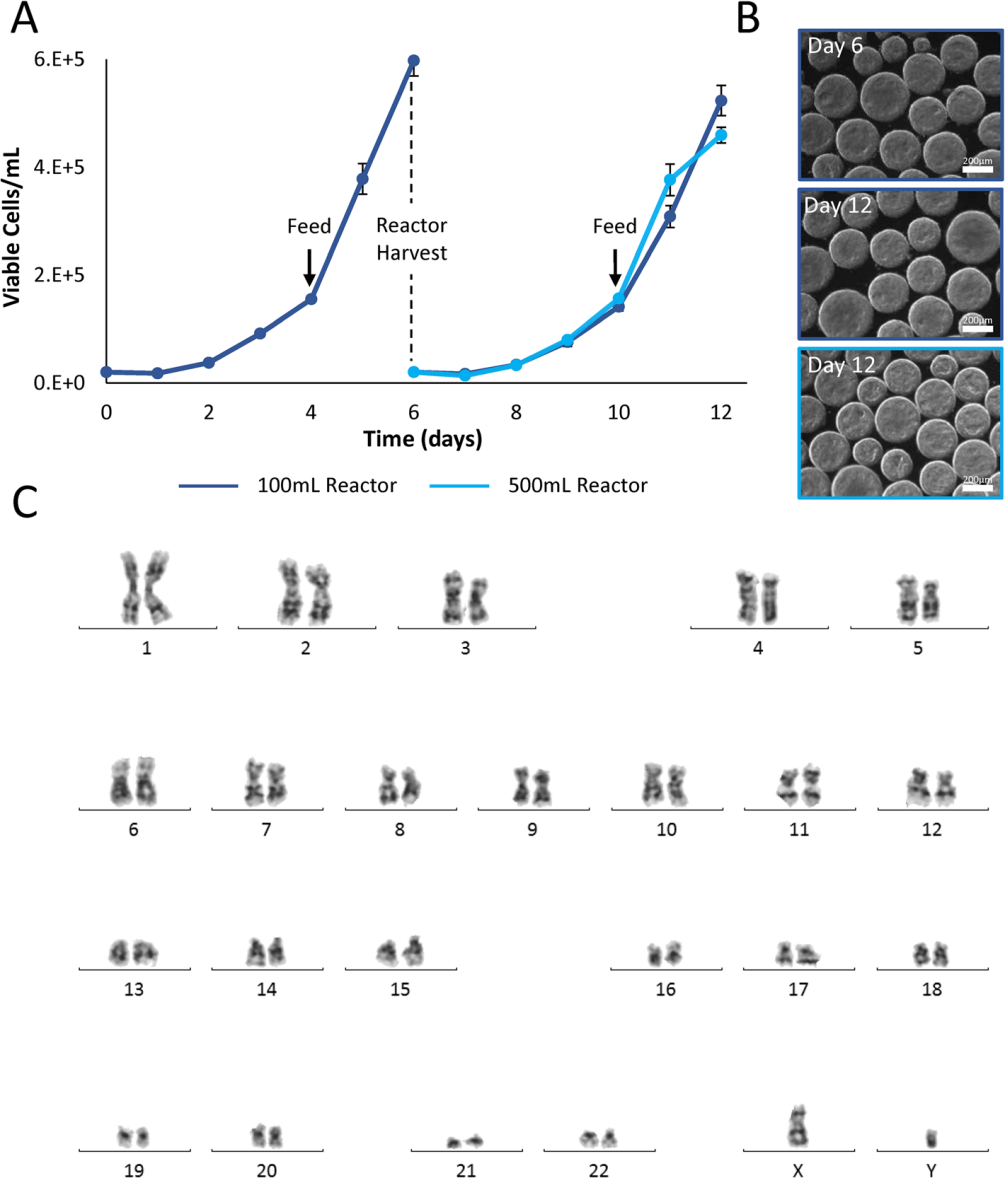
**a** Growth kinetics and **b** representative brightfield microscope images of hiPSCs seeded as single cells cultured in 0.1-L vertical-wheel bioreactors and serially passaged into 0.1-L and 0.5-L vertical-wheel bioreactors (scale bar = 200 μm). **c** Karyogram analysis of hiPSCs taken from the final day of the vertical-wheel bioreactor serial passage (day 12). Cell samples were collected from *n* = 4 stirred suspension bioreactors at each condition. The *P* values were set at 0.05 and all graphs are presented with a ± standard error of the mean (SEM)

Finally, optimized protocols for single-cell inoculation and bioreactor harvesting were combined in a serial passage experiment with cell quality testing performed on the final day of culture. We implemented successes from previous experiments, inoculating single cells into the 100-mL vertical-wheel reactor at 40 rpm to be cultured under fed-batch conditions. On day 6 of culture, a full bioreactor harvest using Accutase for 20 min was performed and single cells were re-seeded into additional 100 mL and scaled-up 500-mL vertical-wheel reactors for another passage. Cell growth (Fig. 6a) and aggregate morphology (Fig. 6b) in the second passage in the 100-mL and 500-mL vertical-wheel bioreactors were comparable to that of the first. Importantly, there was no extended lag phase present in the second bioreactor passage, indicating that the aggregates were still within a healthy growth range and that extended enzyme exposure during the harvest did not impact serial expansion. When cells reach oxygen or nutrient limitations before passaging, they exit the exponential growth phase and enter a plateau or cell death phase. This delay in subculturing can result in a significant decrease in cell fold expansion and stem cell differentiation potential in the next passage, which was not observed in this case [46–48].

**Fig. 7:**
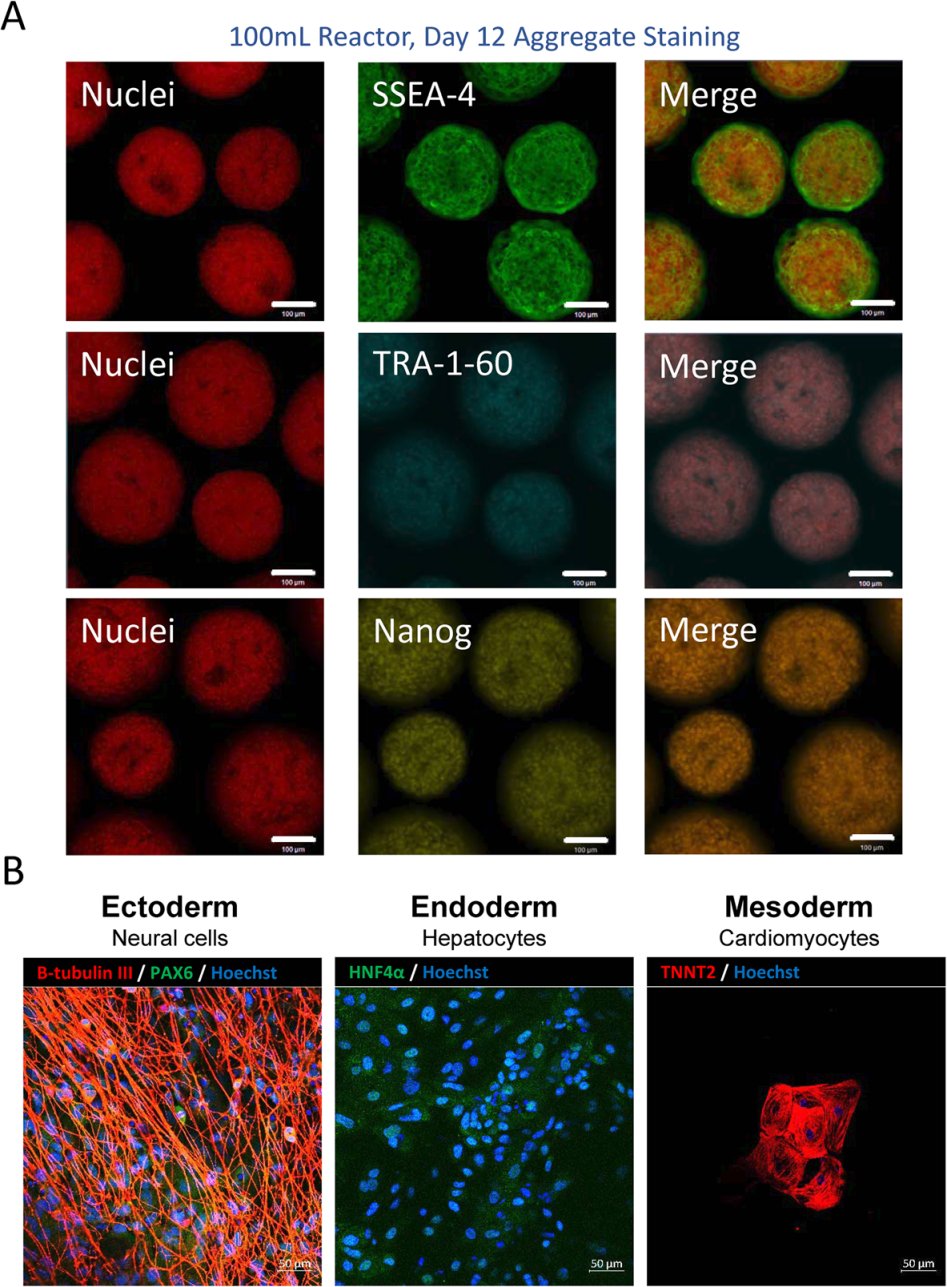
**a** Representative confocal microscope images of hiPSC aggregates taken from the final day of the vertical-wheel bioreactor serial passage (day 12) and stained for pluripotency markers SSEA-4, TRA-1-60, and Nanog (scale bar = 100 μm) and **b** differentiated into neuronal cells (immuno-stained for β-tubulin and PAX6), hepatocytes (immuno-stained for HNF4α), and cardiomyocytes (immuno-stained for TNNT2) (scale bar = 50 μm)

**Fig. 8:**
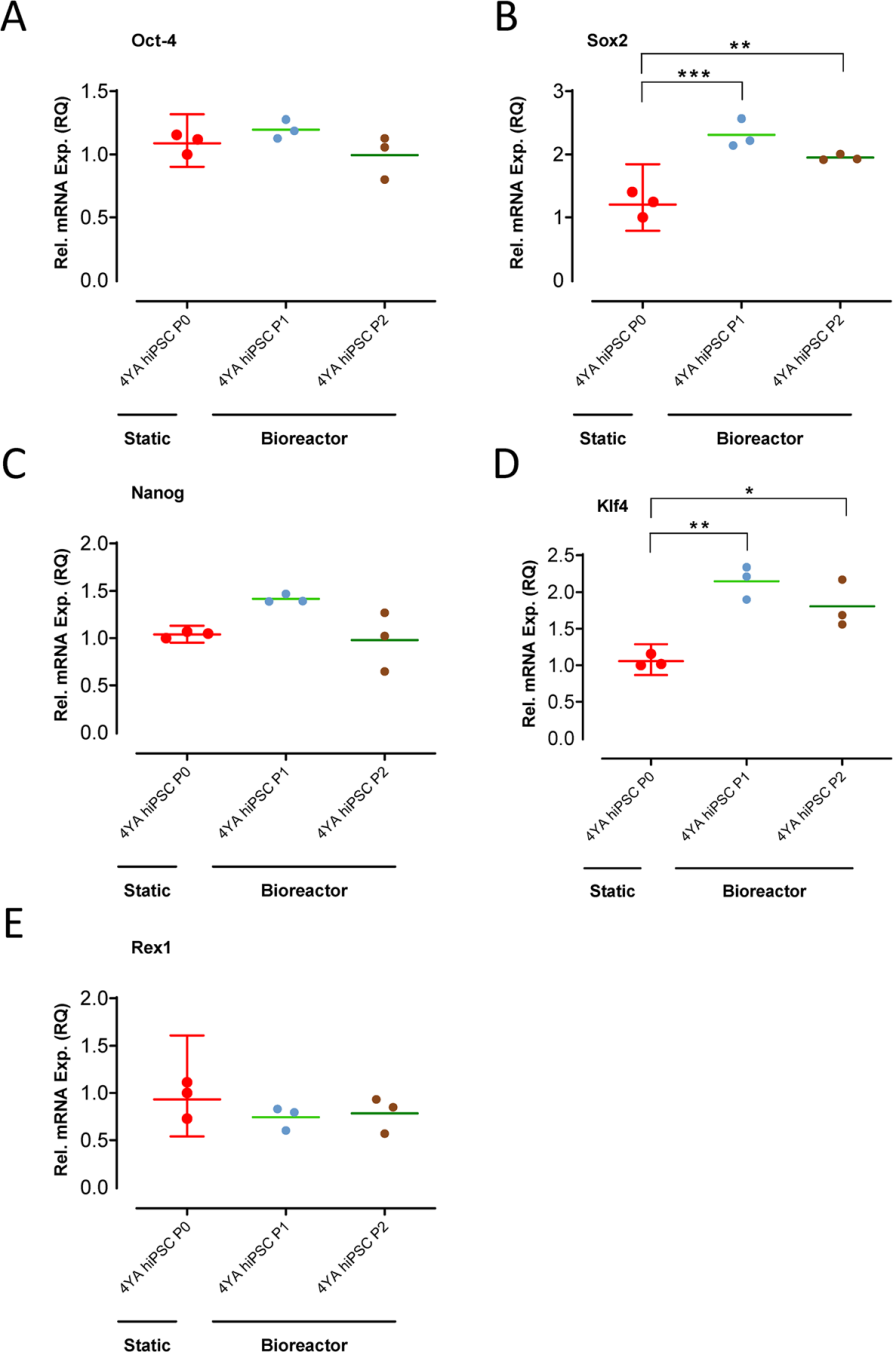
Dotplot geometric mean linear depictions of pluripotency-associated genes **a** Oct-4, **b** Sox2, **c** Nanog, **d** Klf4, and **e** Rex1 expression levels in hiPSCs analyzed by RT-qPCR. hiPSCs cultured in static conditions (P0) were used as a control reference sample. hiPSCs were analyzed following expansion in vertical-wheel bioreactors (P1 = day 6 and P2 = day 12). Expression was quantified relative to the housekeeping gene GAPDH and was normalized to static culture hiPSC level (= 1). RQ relative quantification

Additional samples were collected at the end of the 12-day culture period to assess genomic stability and to determine phenotypic and functional pluripotency quality. Following the 12-day optimized expansion process in the vertical-wheel bioreactors, the hiPSCs presented with a normal chromosome complement (Fig. 6c). hiPSC aggregates maintained expression for human pluripotency markers SSEA-4, TRA-1-60, and Nanog, with confocal slices displaying no difference in spatial expression between the outer edge and the center of the aggregates (Fig. 7a). Directed tri-lineage differentiation into neural cells, hepatocytes, and cardiomyocytes demonstrated that the hiPSCs retained full pluripotency after bioreactor culture, functionally generating cells from the three germ layers (Fig. 7b). Negative control confocal images are included in Supplementary Fig. 1. Pluripotency-associated genes showed either comparable (i.e., Oct-4, Nanog, and Rex1) or higher (i.e., Sox2 and Klf4) expression in hiPSCs following serial passaging in bioreactors compared to their static cultured counterpart (Fig. 8). This corroborates the maintenance of pluripotency state following several passages of hiPSCs in vertical-wheel bioreactors.

## Discussion

The ability of hiPSCs to mimic traditional hESC self-renewal capacity and functional pluripotency potential in vitro and in vivo make them an ideal cell type for generating large quantities of differentiated cells needed for regenerative medicine applications [49]. Epigenetic reprogramming of hiPSCs from somatic cells provides the unique opportunity for personalized regenerative medicine with reduced risks of rejection as well as large-scale disease modeling studies that would otherwise be restricted due to limited availability of primary cells and biopsy material. The transfer of laboratory processes into a manufacturing facility, however, is one of the most critical steps required for the production of cell-based therapies and products and is currently bottlenecked by a lack of scalable bioprocess protocols for production of clinical quantities of high-quality hiPSCs [50].

While stirred-tank bioreactors offer several advantages for stem cell manufacturing, there are major limitations related to the complex hydrodynamics and high shear stress at the impeller tip. Traditional stirred-tank bioreactors employ horizontal-blade or turbine impellers that require careful optimization at each scale. The shear stress at the impeller tip increases with reactor scale, limiting the successful scale-up of shear-sensitive stem cells [51]. The vertical-wheel bioreactor that was hydrodynamically characterized and utilized in this study could prove to be invaluable to overcoming these hurdles for scale-up of hiPSCs, which are among the most difficult cell types to cultivate. Unlike mouse cells, human pluripotent stem cells differentiate extensively when cultured as aggregates in stirred suspension bioreactors [52]. Even compared to traditional hESCs, hiPSCs display slower growth kinetics and impaired directed differentiation, making an optimized culture environment especially important for their successful cultivation [53]. As highlighted by the CFD models generated in this study, the vertical-wheel reactor is unique in its mixing ability, directing fluid streamlines in a lemniscate pattern throughout the entire volume of the reactor. This results in a more uniform distribution of hydrodynamic forces and a lower shear stress environment, ideal for hiPSC growth as aggregates. The hydrodynamic distribution of energy dissipation rate, which scales exponentially with an increase in agitation, is a controlling variable dictating average aggregate size and size distributions [54]. Among the lower agitation rates modeled (40 rpm), the energy dissipation rate remained homogeneous throughout the vertical-wheel volume, leading the authors to believe that hiPSC aggregates could be successfully cultured as single cells in this bioreactor environment.

When single-cell inoculation of hiPSCs was tested in traditional horizontal-blade bioreactors, the authors were unsuccessful in generating consistent aggregate sizes. At all tested agitation rates (40 rpm, 60 rpm, and 80 rpm), aggregate distributions were bi-model or spread out in nature corresponding to low cell yields. This could be a result of the predominantly radial mixing present within the horizontal-blade bioreactor that limits aggregates from moving throughout the entire volume. Our previous work modeling 100-mL horizontal-blade bioreactors highlighted differences in hydrodynamics between the vertical planes in the reactor [33, 54]. Above and below the impeller blade were areas with relatively low hydrodynamic values and fluid dead zones. Conversely, the middle section of the working volume height experienced relatively high hydrodynamic forces. If aggregates in the horizontal-blade reactor flow in a mostly radial fashion along with the fluid, they will become trapped in either a high shear zone (small aggregates) or a low shear zone (large aggregates). Single-cell hiPSC inoculation was successful in the vertical-wheel bioreactors, producing a narrow distribution of aggregate sizes at each agitation rate with high cell fold expansions, particularly at 40 rpm. With a fed-batch feeding strategy employed, over 30-fold expansion in 6 days was achieved, which is significantly higher than other published studies which inoculate hiPSCs in bioreactors using cell clumps or single cells at high cell densities (2 × 10^5^–1 × 10^6^ cells/mL). Early publications achieve a maximum of 6-fold expansion in 4 to 7 days [15], while recent publications achieve a maximum of 10-fold expansion in 12 days [55] and 10- to 16-fold expansion in 7 days [56].

Literature has indeed provided evidence that hiPSCs harvested as single cells are more likely to acquire genetic abnormalities [57]. Conventional methods used to subculture hiPSCs include manual scraping and microdissection as well as enzymatic and non-enzymatic procedures to detach cell clumps from their matrix. Manual methods to select colonies are labor intensive and highly dependent on the proficiency of skilled technical personal, making them infeasible for a manufacturing setting [58]. Passaging of cell clumps lacks standardization in counting and measuring clump sizes, making it impractical for large-scale, reproducible results [50]. Clump sizes are difficult to control resulting in increased heterogeneity in the seeding population [11, 24]. The precise number and spatial coordination of various cell-cell interactions involved in aggregate growth and embryoid body formation influences cell quality and the course of cell differentiation, making a controlled aggregate population an essential consideration [59, 60]. While others have shown that under certain culture conditions single-cell inoculated hiPSC maintenance of pluripotency and karyotype stability is possible [61, 62], to the best of our knowledge, current publications have not investigated bioreactor harvesting of hiPSC aggregates dissociated into single cells for serial passaging. Bioreactor harvesting is an essential step in manufacturing scale-up of stem cell culture but is rarely investigated. The authors were successful in designing a full reactor harvest protocol for the dissociation of hiPSC aggregates into single cells. This involved finding an appropriate enzyme, exposure time, reduced working volume, and agitation rate within the reactor to dissociate aggregates into single cells without sacrificing the cells ability to recover in growth and maintain pluripotent quality characteristics. Recently, bioprocess publications have investigated bioreactor harvesting of mesenchymal stromal cells grown on microcarriers [63–66]. These studies optimized protocols by testing many of the same variables, but recovery yields generally hovered around 80%. This reduced recovery, compared to the 95% achieved in this study, is likely attributed to the additional challenges associated with separating the cells from the microcarriers through a multi-filtration and washing process.

Importantly, the cells harvested and re-inoculated as single cells in both 0.1-L and scaled-up 0.5-L vertical-wheel bioreactors not only maintained consistent growth kinetics, they maintained a normal karyotype and pluripotent function after 12 days of bioreactor culture. While there are a few studies with mouse ESCs cultured on microcarriers [67–69] or as aggregates [70], there lacks a robust method for serial culturing of human ESCs in stirred suspension culture. A recent publication [71] demonstrated that hESCs cultured on microcarriers often lose their stemness through successive passages. In this study, 3-fold expansion was initially achieved over 5 days; however, with each successive passage, cell expansion was reduced until cells could not be passaged. In our recent publication [41], we showed that hiPSCs could be cultured through successive serial passages in the vertical-wheel bioreactor with expansions ranging between 30 and 35-fold each passage. While we were able to show that growth and pluripotency could be maintained in the bioreactors, the protocol required pre-formed aggregate inoculation and a cell sample passaging protocol, limiting our ability to scale-up the process. Additionally, it was noted that inconsistent lag phases resulted between passages, such that culture periods would range between 6 and 8 days. These inconsistencies in passage length are not acceptable in a manufacturing setting. By utilizing single-cell inoculation and the full reactor harvest protocol optimized in this study, the increased lag phase was not observed in the serial passage. The hiPSCs generated in the vertical-wheel reactor maintained high-quality standards in the generation of morphologically healthy and homogenous aggregates. The cells maintained a normal karyotype, an expression of characteristic pluripotency markers, and the ability to differentiate into cell types of all three germ layers. It should be noted that the tri-lineage differentiation performed in this study was of a qualitative nature. There are numerous bioprocess challenges associated with obtaining high differentiation efficiencies which the authors hope to address in future studies. The methods optimized in this study for generation of large quantities of high-quality hiPSCs in the vertical-wheel bioreactor overcome some of the major bottlenecks in moving production to the clinical and manufacturing setting.

## Conclusions

hiPSCs carry enormous promise for breakthroughs in understanding human development, drug screening, disease modeling, and cell and gene therapies; however, their therapeutic potential has been bottlenecked in a laboratory setting due to bioprocess challenges in scale-up of large quantities of high-quality cells. This study focused on characterizing and optimizing the use of vertical-wheel bioreactors as a tool to overcome hiPSC production challenges. The unique bioreactor geometry provided a low shear stress environment with a more homogeneous distribution of hydrodynamic forces. While a single-cell hiPSC inoculation method proved unsuccessful in traditional horizontal-blade bioreactors, the vertical-wheel environment supported healthy morphological aggregate growth, high cell fold expansions, and maintenance of pluripotency cell quality. This study also includes the first published protocol for in-vessel hiPSC aggregate harvesting, permitting the entire bioreactor volume to be dissociated into single cells for serial passaging and scale-up. These protocols provide a feasible solution for the culture of high-quality hiPSCs at a clinical and manufacturing scale by overcoming some of the major documented bioprocess bottlenecks.

## Supplementary Information

**Additional file 1: Supplementary Table 1.** Primer sequences used for RT-qPCR analysis.

**Additional file 2: Supplementary Fig. 1.** Representative confocal images are shown for negative control staining. Scale bars = 50 μm.

## Data Availability

Not applicable

## Abbreviations

CAD: Computer-aided design
CFD: Computational fluid dynamics
CFL: Courant-Friedrich-Lewy
hiPSC: Human induced pluripotent stem cell
PBS: Phosphate buffer solution
PIV: Particle image velocimetry
rpm: Revolution per minute
RT-qPCR: Reverse transcription quantitative polymerase chain reaction
RQ: Relative quantification
SEM: Standard error of the mean
SIMPLE: Semi-implicit method for pressure-linked equations
