# Supplementary figures and images for: Overcoming bioprocess bottlenecks in the large-scale expansion of high-quality hiPSC aggregates in vertical-wheel stirred suspension bioreactors
Source: Stem Cell Res Ther. 2021 Jan 13;12:55. doi: 10.1186/s13287-020-02109-4 (PMC7805206; doi:10.1186/s13287-020-02109-4)

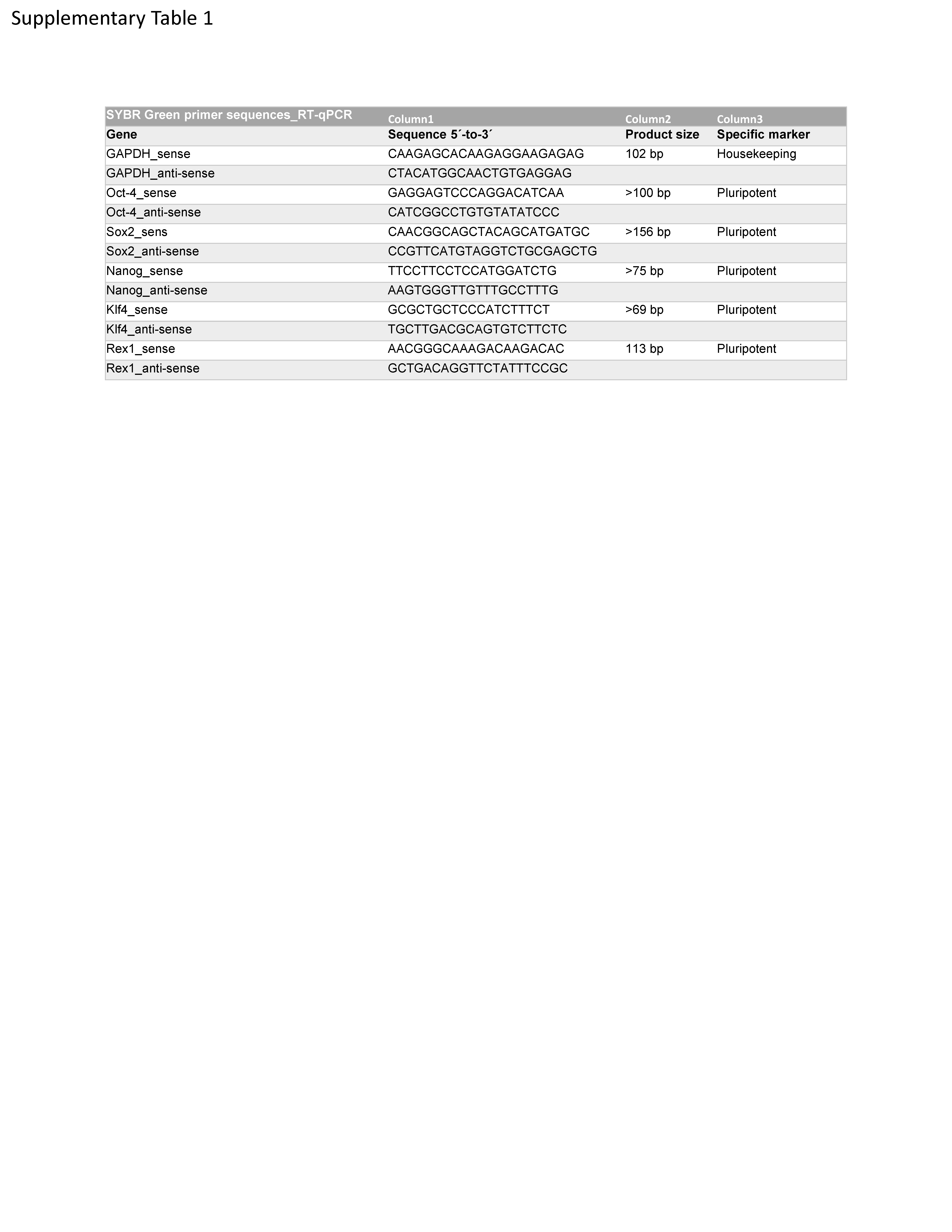

Supplement: Supplementary file 1 — Additional file 1: Supplementary Table 1. Primer sequences used for RT-qPCR analysis. [file 13287_2020_2109_MOESM1_ESM.tiff]

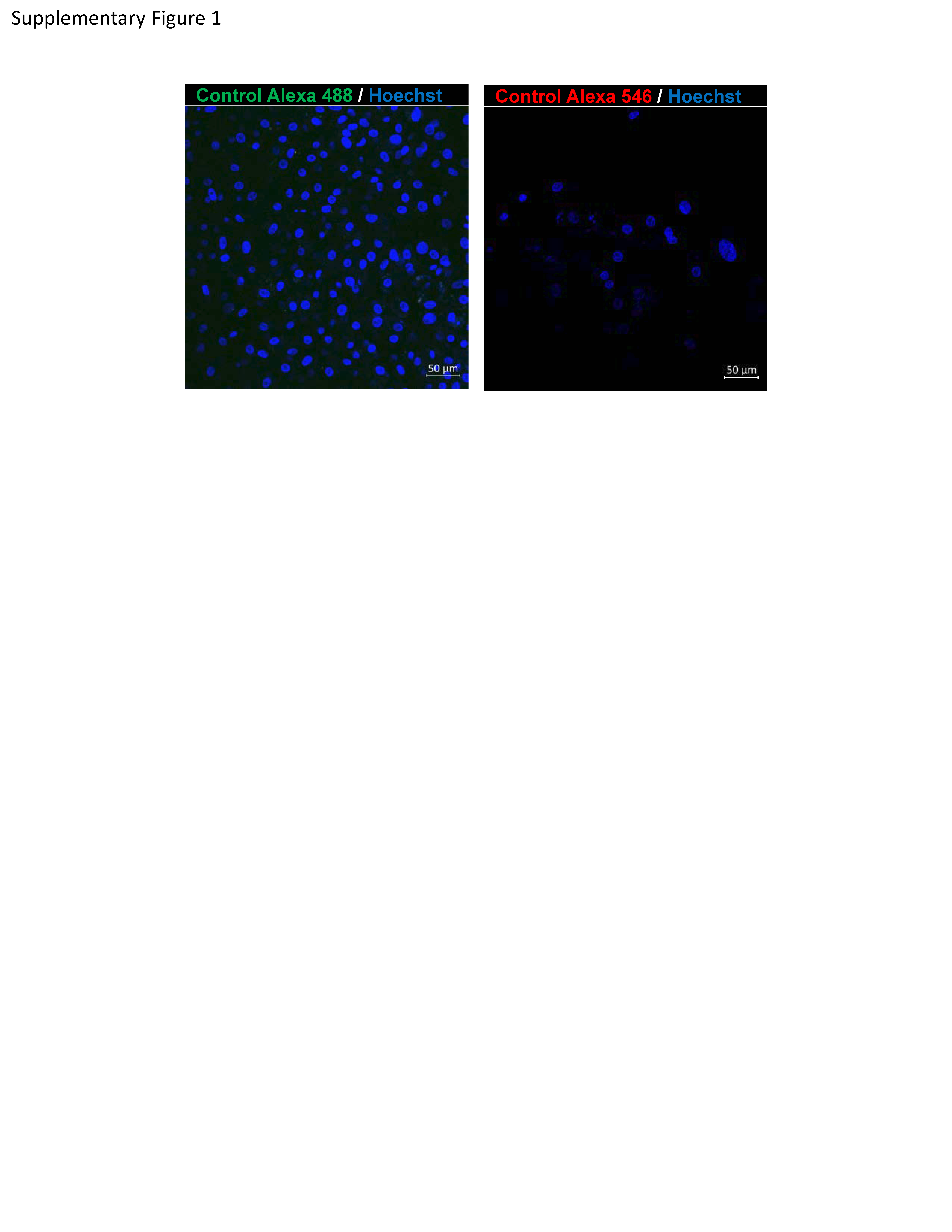

Supplement: Supplementary file 2 — Additional file 2: Supplementary Fig. 1. Representative confocal images are shown for negative control staining. Scale bars = 50 μm. [file 13287_2020_2109_MOESM2_ESM.tiff]
